# Effects of LncRNA MEG3 on immunity and autophagy of non-small cell lung carcinoma through IDO signaling pathway

**DOI:** 10.1186/s12957-021-02346-8

**Published:** 2021-08-16

**Authors:** Chuanqiang Wang, Xiangbo Tao, Jungong Wei

**Affiliations:** 1 Cardiothoracic Surgery Department, Shandong Guoxin Healthcare Group Zibo Hospital, Shandong, 255120 China; 2 Department of Oncology, Chengwu County People’s Hospital, Shandong, 274200 China

**Keywords:** LncRNA MEG3, IDO signaling pathway, Non-small cell lung carcinoma, Immunity, Autophagy

## Abstract

**Background:**

The study was done to investigate the effect of LncRNA MEG3 on the immunity and autophagy of non-small cell lung carcinoma through the IDO signaling pathway.

**Methods:**

A total of 78 cases of early NSCLC patients (research group; RG) and 69 cases of health controls (control group; CG) during the same time were included. The contents of LncRNA MEG3 and miR-543 in peripheral blood and tissues and their diagnostic values for NSCLC were detected. The relationship between LncRNA MEG3 and miR-543 and their posttreatment contents and influence on the prognosis of NSCLC patients were tested. The expression of LncRNA MEG3, miR-543, and IDO (IDO1, IDO2, and TDO proteins) in the lung tissue of rats and the immune function in the CG and the RG were detected. The effects of LncRNA MEG3 and miR-543 on the biological behavior of NSCLC cells were determined. The role of LncRNA MEG3, miR-543, and IDO in NSCLC was verified.

**Results:**

LncRNA MEG3 was low in peripheral blood and tissues, while miR-543 was high (*P* < 0.05); both had good diagnostic values for NSCLC (*P* < 0.05). LncRNA MEG3 had a negative correlation with miR-543 (*P* < 0.05) and influenced the prognosis of NSCLC patients (*P* < 0.05). LncRNA MEG3 in the lung tissue of rats using IDO inhibitor was elevated compared with that of lung carcinoma model rats (*P* < 0.05). The level of miR-543 was declined compared with that of lung carcinoma model rats (*P* < 0.05). The levels of IDO1, IDO2, and TDO proteins were evidently declined compared with those of lung carcinoma model rats (*P* < 0.05). Compared with lung carcinoma model rats, CD3^+^, CD4^+^, and CD4^+^/CD8^+^ of IDO inhibitor rats were elevated, while CD8^+^ was declined (*P* < 0.05). Cell proliferation and invasion ability and IDO1, IDO2, TDO, Beclin-1, and LC3-II proteins were declined in the sh-LncRNA MEG3 group (*P* < 0.05), while those in the mimics-miR-543 group were evidently elevated (*P* < 0.05). However, the double luciferase activity detection and RIP experiment confirmed that there was targeted regulation among them (*P* < 0.05).

**Conclusion:**

MEG3 has low expression in NSCLC and affects the immunity and autophagy of NSCLC cells via regulating the miR-543/IDO signaling pathway, which is effective for the treatment of NSCLC.

## Introduction

Lung carcinoma originated from the bronchial mucosa or gland of lung, which is a common malignancy in the clinic [1], with a mortality ranking the first among malignant tumors [2]. According to the histopathological characteristics, it is grouped into non-small cell lung carcinoma (NSCLC) and small cell carcinoma [3]. NSCLC accounts for about 85% of lung carcinoma cases [4]. However, due to the lack of special and remarkable symptoms in the early phase of NSCLC, most patients were confirmed until the disease had developed to the middle and late stages, which leads to missed optimal timing for operation and susceptibility to recurrence after operation, greatly reducing the survival rate of patients [5]. At present, the etiology of lung carcinoma is still under exploration. The pathogenic factors mainly include smoking, occupational exposure, ionizing radiation, heredity, and history of lung disease [6]. The main treatment for NSCLC is surgery combined with radiotherapy and chemotherapy [7]. But its 5-year survival rate is still not ideal. In recent years, with the development of molecular science, tumor treatment has made rapid progress, which brings good news to NSCLC patients, especially advanced patients [8].

LncRNA, a RNA with more than 200 nucleic acids, has the specificity of development and tissue expression and is abnormal in many carcinomas and participates in post-transcriptional shearing, editing, transportation, translation, and degradation [9, 10]. Many reports have confirmed that LncRNAs have the potential as molecular markers for tumor diagnosis and prognosis [11, 12]. The maternally expressed gene 3 (MEG3), highly expressed in normal tissues, is an imprinted gene located at 14q32.3 [13]. Xu J and Xu Y suggest that LncRNA MEG3 acts on the progression of osteoarthritis [14]. Zhang et al. show that LncRNA MEG3 has a correlation with the occurrence of laryngeal squamous cell carcinoma and leukemia [15]. However, referring to the previous data, we found little information regarding the role of LncRNA MEG3 in NSCLC. Therefore, we tested the role of LncRNA MEG3 in NSCLC, so as to provide new ideas for the treatment of NSCLC.

## Materials and methods

### Research participants

#### Data of patients

From May 2016 to May 2018, 78 patients with early NSCLC (research group, RG) and 69 healthy individuals (control group, CG) were included as research participants. This research conformed to the guidelines of the Ethics Committee and was agreed by the patients and their families.

#### Inclusion and exclusion criteria

For the inclusion criteria, the patients were confirmed as NSCLC after biopsy in the laboratory, imaging, and pathology department of our hospital; all participants had complete data; the informed consent was obtained from the patients or their family members.

Exclusion criteria included those with other tumor diseases or major organ damage, those who have been treated, those with immune diseases, those with low treatment compliance, those with drug allergy, and referred cases.

Inclusion criteria of the CG included healthy people who underwent physical examination in our hospital and patients without major medical history before; all the results of the physical check-ups were normal; the informed consent was obtained.

Note: There was no statistical difference in the general data, including age, BMI, smoking, drinking, living environment, and nationality between the two groups (*P* > 0.05).

#### Collection of human samples

Peripheral blood (5 ml) was drawn from patients in the morning after admission, and sent to the laboratory by the medical staff for centrifugation at 4 ℃ with 1505 × *g*. The extracted serum was placed in a refrigerator at – 80 ℃. The carcinoma tissues and adjacent tissues removed during pathological biopsy were embedded in paraffin and sliced with a thickness of 4 μm. The sections were then sealed with goat serum and added with primary antibody and secondary antibody diluted at 1:100, followed by 6–8 min of dyeing with digital audio broadcasting (DAB) staining solution and 10 s of re-dyeing with hematoxylin.

### Data of rats

Sixty SPF C57BL/6 male rats were obtained from Charles River (certificate number SCXK (Beijing) 2016–0011). The rats were aged 12 weeks and weighed 200–250 g.

#### Rat modeling

Forty rats were randomly selected to establish the lung carcinoma model. Walker-256 carcinoma cells were cultured in RMPI-1640 including 10% FBS. Walker-256 cells were adjusted to 1 × 10 cells/ml and inoculated into the abdominal cavity of KM mice with 0.2 ml/cell. After ascites appeared, ascites of Walker-256 cells of KM mice were extracted and diluted with normal saline. The cell density was adjusted to 0.5 × 10 cells/ml. SD rats were injected with ascites cell suspension at 0.2 ml/rat via caudal vein, and fed continuously for 3 weeks. After imaging examination, the formation of lung tumor in all rats was regarded as successful modeling. The other 20 rats were kept without treatment.

#### Treatment for different groups

Lung carcinoma model rats were grouped. One group was injected with 6 mg/ml indoleamine-2,3-dioxygenase (IDO) inhibitor (NLG919) into the lung tissue of rats for 48 h. The other group was treated with the same amount of normal saline for 48 h.

#### Sample collection

All rats were killed by cutting their necks, and their lung tissues were obtained. The tissues were embedded in paraffin and sliced with a thickness of 4 μm. The sections were then sealed with goat serum, and added with primary antibody and secondary antibody diluted at 1:100, followed by 6–8 min of dyeing with DAB staining solution and 10 s of re-dyeing with hematoxylin.

### Cell data and culture

Human NSCLC cells HCC827 (BNCC102152), A549 (BNCC100258), NCI-H1299 (BNCC100268), H125 (BNCC100831) and human alveolar epithelial cells HPAEpiC (BNCC337859) were purchased from ATCC. A DMEM medium containing 10% FBS and 1% penicillin/streptomycin mixture was applied for cultivation at 37 ℃ with 5% CO_2_. When the cells reached 80% fusion degree, they were digested with trypsin, and the medium was replaced according to the ratio of 1:3, and then they were passaged.

#### PCR testing

Total RNA was obtained using Trizol total RNA extraction kit, and the purity of total RNA was tested using ultraviolet spectrophotometer. Total RNA was reverse transcribed into cDNA for PCR detection. Reaction conditions are as follow: 95 ℃ for 1 min, 92 ℃ for 30 s, 92 ℃ for 30 s, 58 ℃ for 30 s, 73 ℃ for 30 s, with a total of 38 times. Each sample was given 3 repeated wells. The data were tested using 2^−△△ct^ (Table 1).

**Table 1:** Primer sequence

	Upstream primer 5'-3	Downstream primer 5'-3'
lncRNA MEG3	CTGCCCATCTACACCT CACG	CTCTCCGCCGTCTGCGCTAGG GGCT
GAPDH	CTCGCT TCGGCAGCACATATACT	ACGCTT CACGAATTTGCGTGTC
miR-543	CCAGCTACACTGGGCAGCAGCAATTCATGTTT	CTCAACTGGTGTCGTGGA
U6	CTCGCTTCGGCAGCACATATACT	ACGCTTCACGAATTTGCGTGTC

#### Cell transfection

LncRNA MEG3 and miR-543 in each cell line were detected, and the two with the most significant difference were obtained for subsequent tests. LncRNA MEG3 knockdown (si-LncRNA MEG3), LncRNA MEG3 overexpression (sh-LncRNA MEG3), and negative control (NC) and miR-543 knockdown, miR-543 overexpression, and negative control (NC-miR) were transfected into cells using Lipofectamine™ 2000. After transfection for 6 h, the culture medium was replaced for 48 h, and the transfection efficiency was tested using qRT-PCR.

#### MTT assay

After the 24-h transfection, the cells were seeded on a 96-well plate and incubated at 37 ℃ for 24 h, 48 h, 72 h, and 96 h, respectively. A 20-μl MTT solution (5 μg/ml) was added at the abovementioned time points. After 4 h, the supernatant was discarded, and 150 μl dimethyl sulfoxide (DMSO) was put into each well and shaken sufficiently to dissolve the crystals. The absorbance at 490 nm was tested.

#### Transwell test

The treated cells were inoculated into a 24-well plate, adjusted to 3 × 10^4^ cells/well, and digested with trypsin. Then, they were moved to the upper chamber where 200 μl RPMI1640 culture medium was added, while the lower chamber was added with 500 ml RPMI1640. After 48 h of cultivation at 37 ℃, the small chamber was taken out to discard the cells in the upper chamber, while the remaining cells were fixed with methanol for 30 min after PBS washing, dyed with 0.1% crystal violet for 20 min and rinsed with PBS.

#### Western blot detection

RIPA buffer was used to extract proteins from cultured cells of each group, and the lysate was centrifuged at 10,000 × *g* for 20 min to obtain the supernatant. The protein concentration was tested using the BCA method. The same amount of protein was separated for SDS-PAGE and membrane transfer, and sealed with 5% skimmed milk powder at room temperature for 1 h. After electrophoresis, the protein was moved to PVDF, which was sealed with 5% skim milk for 2 h, rinsed, and cultivated with I antibody (1:1000) overnight at 4 ℃. The primary antibody was removed, and horseradish peroxidase-labeled goat anti-rabbit secondary antibody was applied and then exposed.

#### Double fluorescein reporter enzyme

Luciferase reporter vectors [psiCHECK2-MEG3 wild type (WT), psiCHECK2-MEG3 mutant (MUT)] and mimics-miR-543 or miR-543-inhibition were treated with NSCLC cells using the lipofectamine method. After 5 h of culture, fresh culture medium was applied, and the transfection was lasted for 48 h. The ratio of the luminous intensity of sea cucumber luciferase to firefly luciferase represented the binding force between miR-543 and LncRNA MEG3.

#### Immunocoprecipitation detection

NSCLC cells were lysed using EZMagna RIP kit and cultivated with protein A magnetic beads, which were then treated with antibodies at 4 ℃. After 6 h, the beads were rinsed and then cultivated with 0.1% of SDS or 0.5 mg/ml of protease K at 55 ℃ for 30 min to discard proteins. At last, quantitative reverse transcription polymerase chain reaction (qRT-PCR) was conducted on immunoprecipitated RNA to prove the existence of LncRNA MEG3 and miR-543.

#### RNA pull-down experiment

NSCLC cells were treated with biotinylated miR-543-wt, miR-543-mut, and NC. After 48 h, the cell lysate was cultivated with the probe and M-280 Streptomyces at room temperature. After washing, RNA complexes bound to beads were eluted and extracted to determine LncRNA MEG3 expression by qPCR.

### Outcome measures

The following are the outcome measures:

1. LncRNA MEG3 and miR-543 in the CG and the RG; diagnostic value of LncRNA MEG3 and miR-543 for NSCLC, the correlation of LncRNA MEG3 with miR-543, LncRNA MEG3 and miR-543 in peripheral blood before and after treatment, and the effect of LncRNA MEG3 and miR-543 on the prognosis of patients
2. LncRNA MEG3, miR-543, and IDO (IDO1, IDO2, and TDO proteins) in lung tissues and immune function in both groups
3. The effect of LncRNA MEG3 and miR-543 on the biological behavior of NSCLC cells
4. The correlation among LncRNA MEG3, miR-543, and IDO in NSCLC

### Statistical methods

SPSS24.0 was applied for data analysis and Graphpad8 for figure visualization. The counting data were represented in percentage (%) and compared by chi-squared test. The measurement data were represented as mean ± SD and compared by *t* test. One-way ANOVA and LSD post hoc test were applied for multi-group comparisons. Repeated measurement variance analysis and Bonferroni post hoc test were applied for multiple time point comparisons. The diagnostic value was tested using receiver operating characteristic (ROC) curve. The survival was tested using the Kaplan–Meier method and compared using Log-rank test. *P* < 0.050 indicates a statistically significant difference.

## Results

### Clinical significance of LncRNA MEG3 and miR-543 in NSCLC

**Fig. 1:**
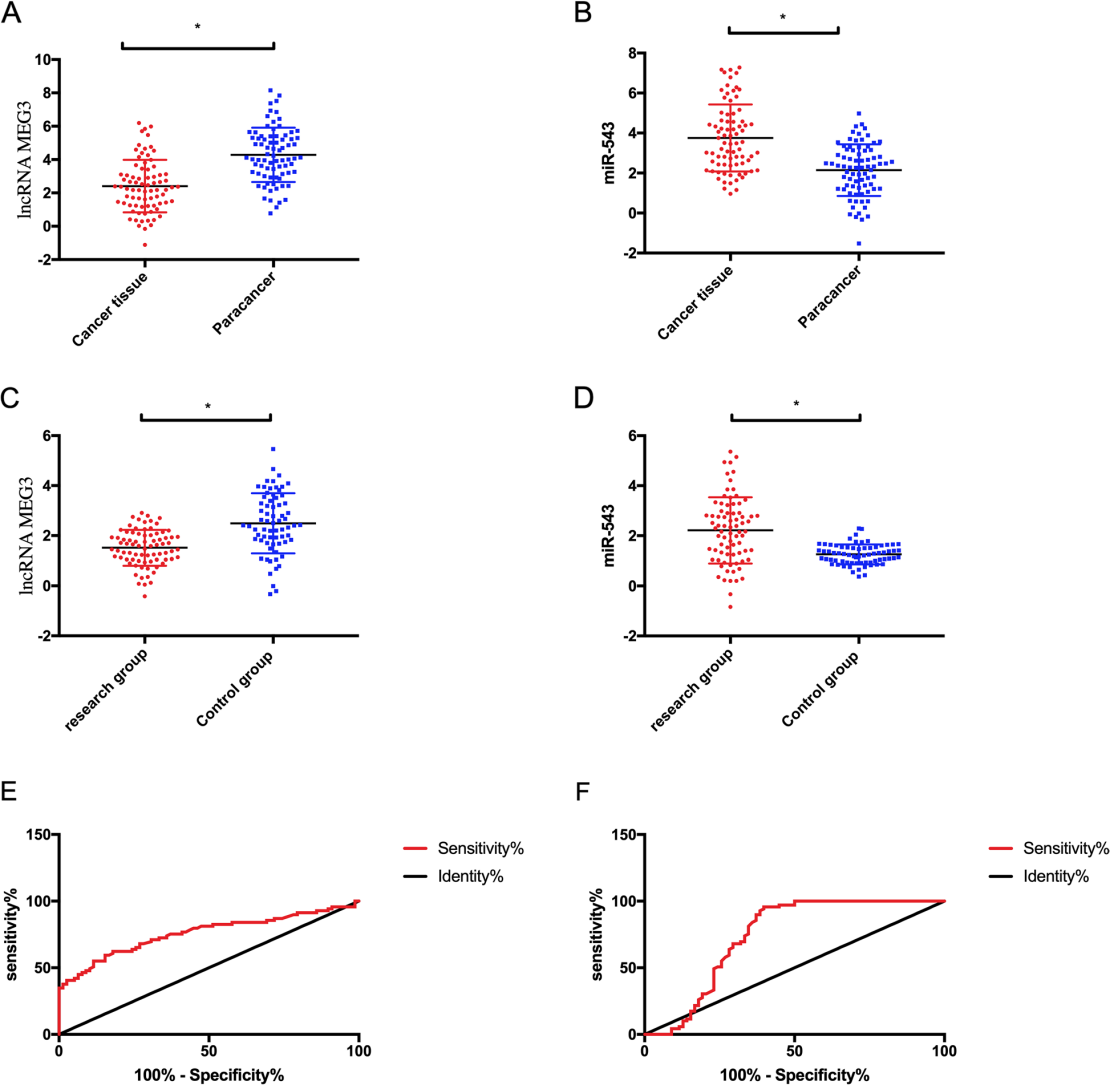
Clinical significance of LncRNA MEG3 and miR-543 in NSCLC. **A** Expression of LncRNA MEG3 in carcinoma tissues and adjacent tissues. **B** Expression of miR-543 in carcinoma tissues and adjacent tissues. **C** Expression of LncRNA MEG3 in two groups. **D** Expression of miR-543 in both groups. **E** ROC curve of LncRNA MEG3 for predicting NSCLC. **F** ROC curve of miR-543 for predicting NSCLC

LncRNA MEG3 and miR-543 in carcinoma tissues and adjacent tissues in the RG were detected. Compared with adjacent tissues, LncRNA MEG3 in carcinoma tissues was evidently declined (*P* < 0.05), and miR-543 was evidently elevated (*P* < 0.05). LncRNA MEG3 in the RG was evidently declined compared with that in the CG (*P* < 0.05), while miR-543 in the RG was evidently elevated compared with that in the CG (*P* < 0.05). The ROC curve revealed that when cutoff value was 2.195, the sensitivity and specificity of LncRNA MEG3 were 60.87% and 82.05%, and when cutoff value was 1.955, the sensitivity and specificity of miR-543 were 95.65% and 58.97% (Fig. 1).

### Analysis of correlation of LncRNA MEG3 with miR-543

**Fig. 2:**
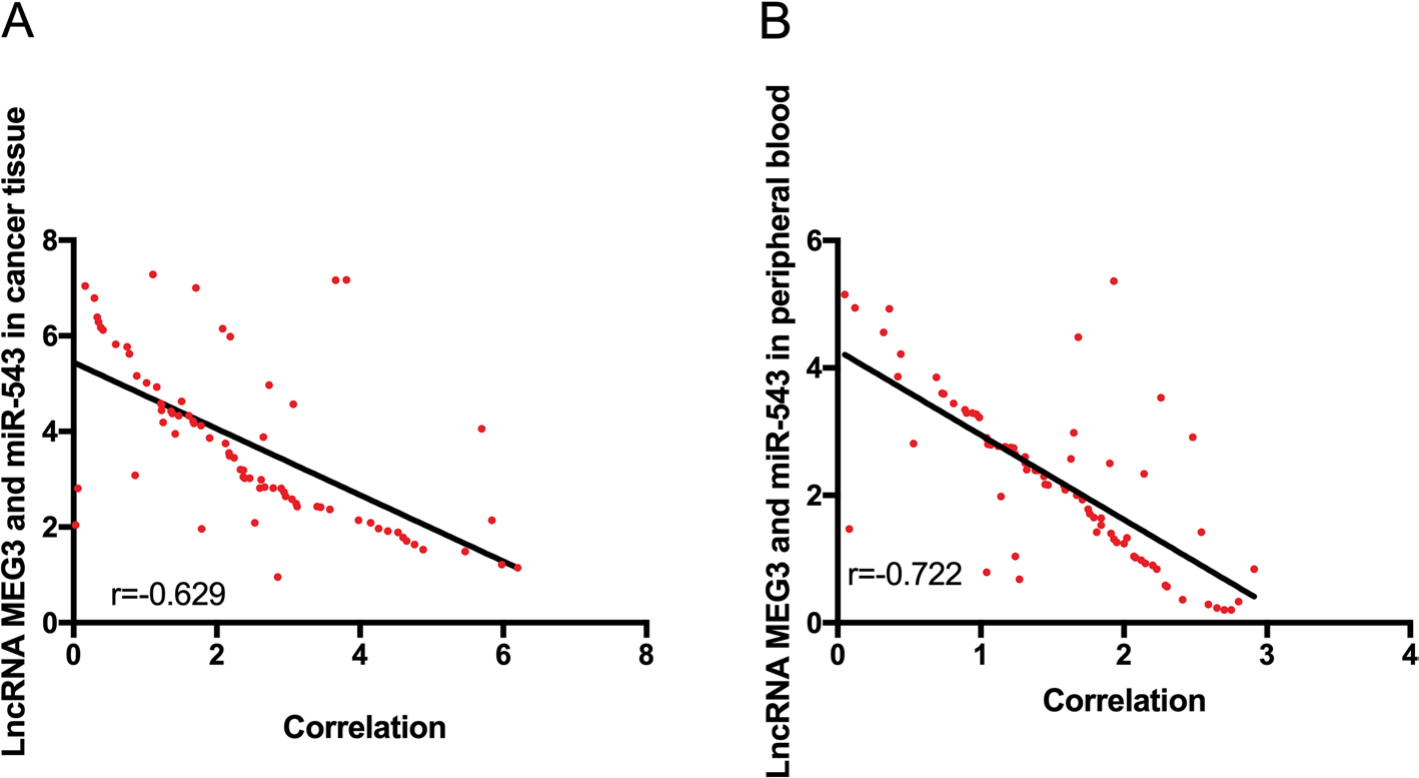
Correlation analysis between LncRNA MEG3 and miR-543. **A** Correlation between LncRNA MEG3 and miR-543 in carcinoma tissues of patients. **B** Correlation between LncRNA MEG3 and miR-543 in peripheral blood of patients

The correlation of LncRNA MEG3 with miR-543 in carcinoma tissues was analyzed. It was found that LncRNA MEG3 was negatively correlated with miR-543 (*P* < 0.05) (Fig. 2).

### LncRNA MEG3 and miR-543 in patients in the RG before and after treatment

**Fig. 3:**
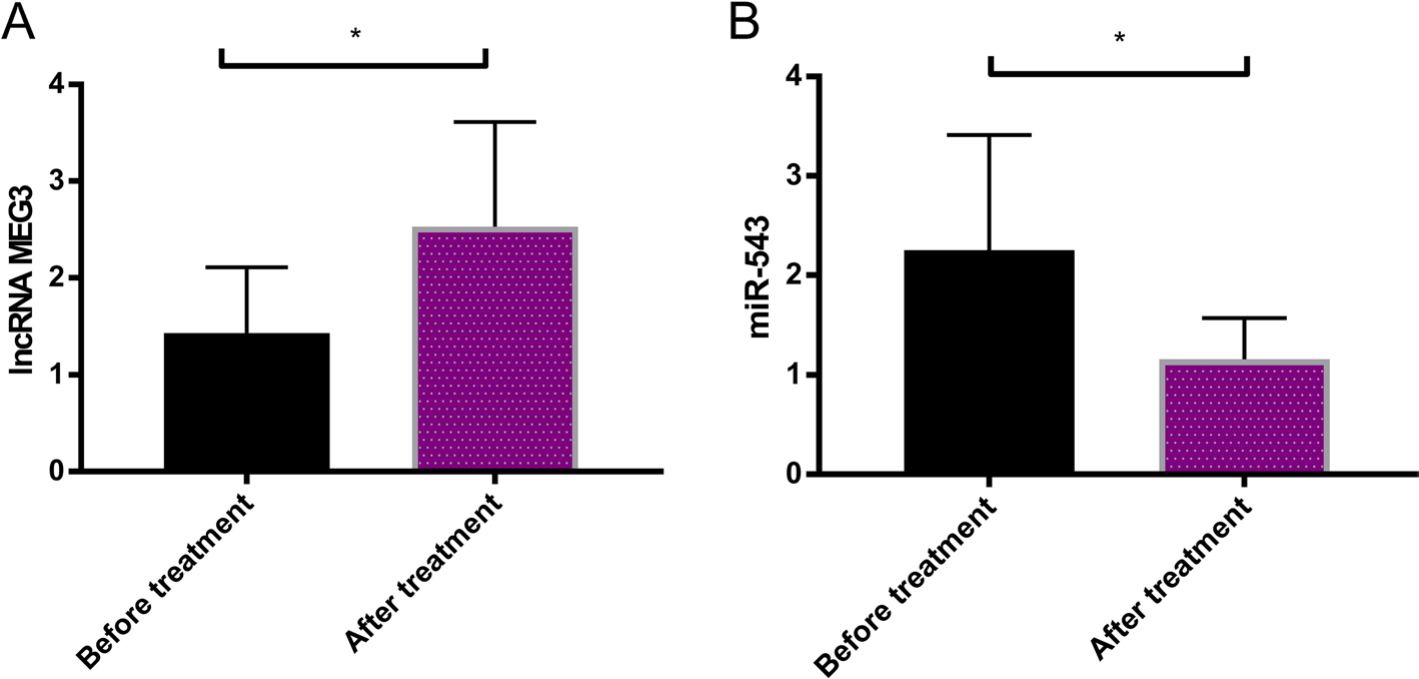
Expression of LncRNA MEG3 and miR-543 before and after treatment. **A** LncRNA MEG3 expression in the RG before and after treatment. **B** miR-543 expression in the RG before and after treatment

LncRNA MEG3 and miR-543 was detected before and after treatment. The results showed that LncRNA MEG3 increased evidently after treatment (*P* < 0.05), while miR-543 decreased evidently after treatment (*P* < 0.05) (Fig. 3).

### Effect of LncRNA MEG3 and miR-543 on the prognosis of NSCLC

**Fig. 4:**
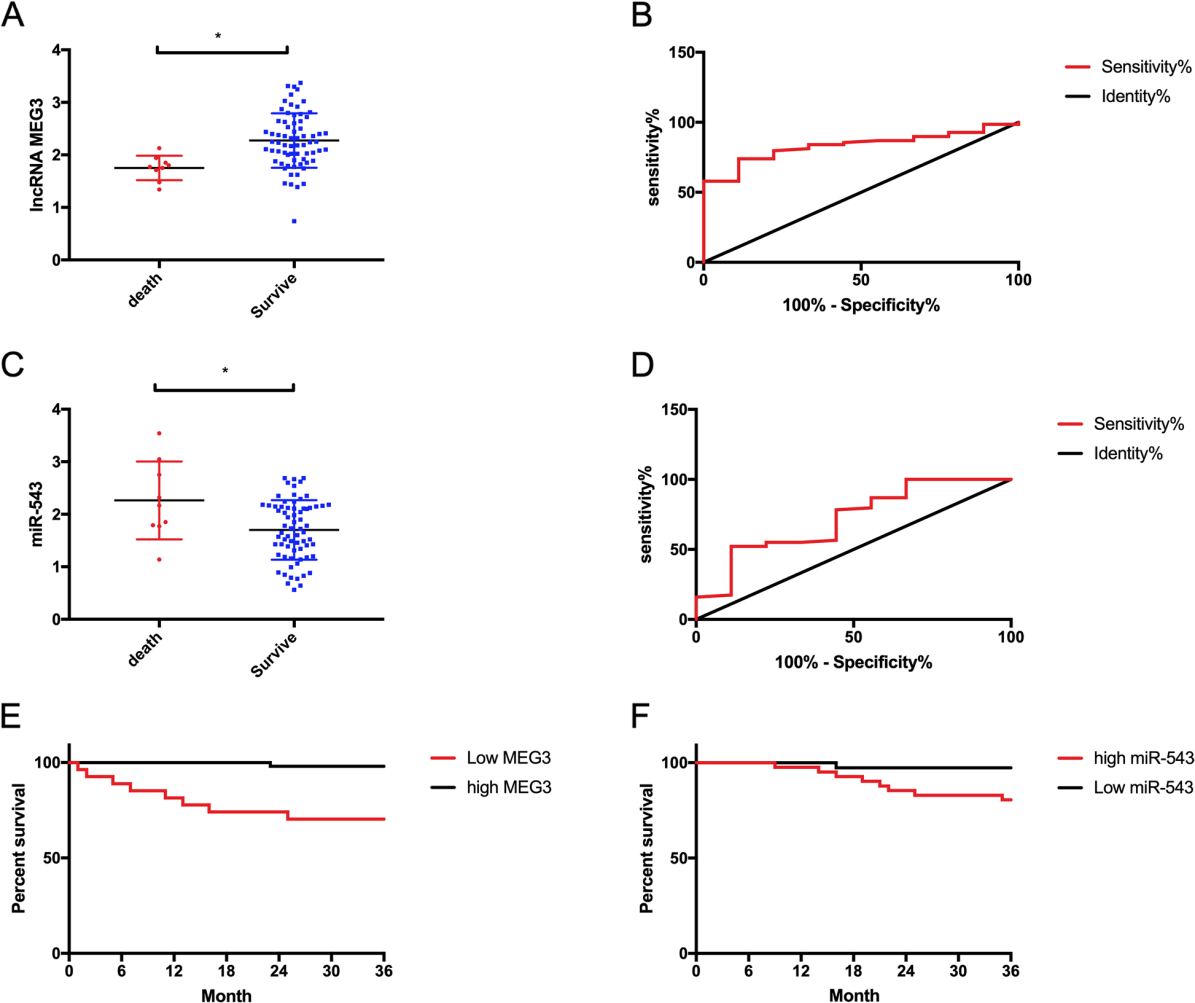
Effect of LncRNA MEG3 and miR-543 on prognosis of NSCLC. **A** LncRNA MEG3 expression in living and dead patients. **B** ROC curve of LncRNA MEG3 for predicting NSCLC mortality. **C** miR-543 expression in living and dead patients. **D** ROC curve of miR-543 in predicting the mortality of NSCLC. **E** Prognosis and survival curves of high and low LncRNA MEG3 groups. **F** Prognosis and survival curves of high and low miR-543 groups

The prognosis of patients was followed up for 3 years, and the follow-up rate was 100%. Nine died, and 69 survived. LncRNA MEG3 and miR-543 in dead patients and alive patients were tested. The testes revealed evidently declined LncRNA MEG3 and significantly elevated miR-543 in dead patients compared with alive patients (*P* < 0.05). According to the cutoff value in ROC, they were divided into the high LncRNA MEG3 group (LncRNA MEG3 ≥ 2.01, *n* = 51), the low LncRNA MEG3 group (LncRNA MEG3 < 2.01, *n* = 27), the high miR-543 group (miR-543 > 1.75, *n* = 41), and the low miR-543 group (miR-543 ≤ 1.75, *n* = 37). The result showed that the prognosis of high LncRNA MEG3 was better than that of low LncRNA MEG3 (*P* < 0.05), and the prognosis of low LncRNA MEG3 was better than that of high LncRNA MEG3 (*P* < 0.05) (Fig. 4).

### Modeling results

Thirty eight of the 40 rats were successfully modeled. After autopsy, it was found that one of the two rats failed to model and was obviously traumatized; this was presumed to be caused by the attack of caged animals, while the other was emaciated, and there was no food residue in the stomach. Therefore, it was presumed that the rats had no ability to eat independently. No peritonitis occurred in rats in this experiment. Among them, 20 rats were raised normally, 19 rats were lung carcinoma model rats, and 19 rats were treated with IDO inhibitor.

### Detection of LncRNA MEG3, miR-543, and IDO (IDO1, IDO2, and TDO proteins) in lung tissue of the three groups of rats

**Fig. 5:**
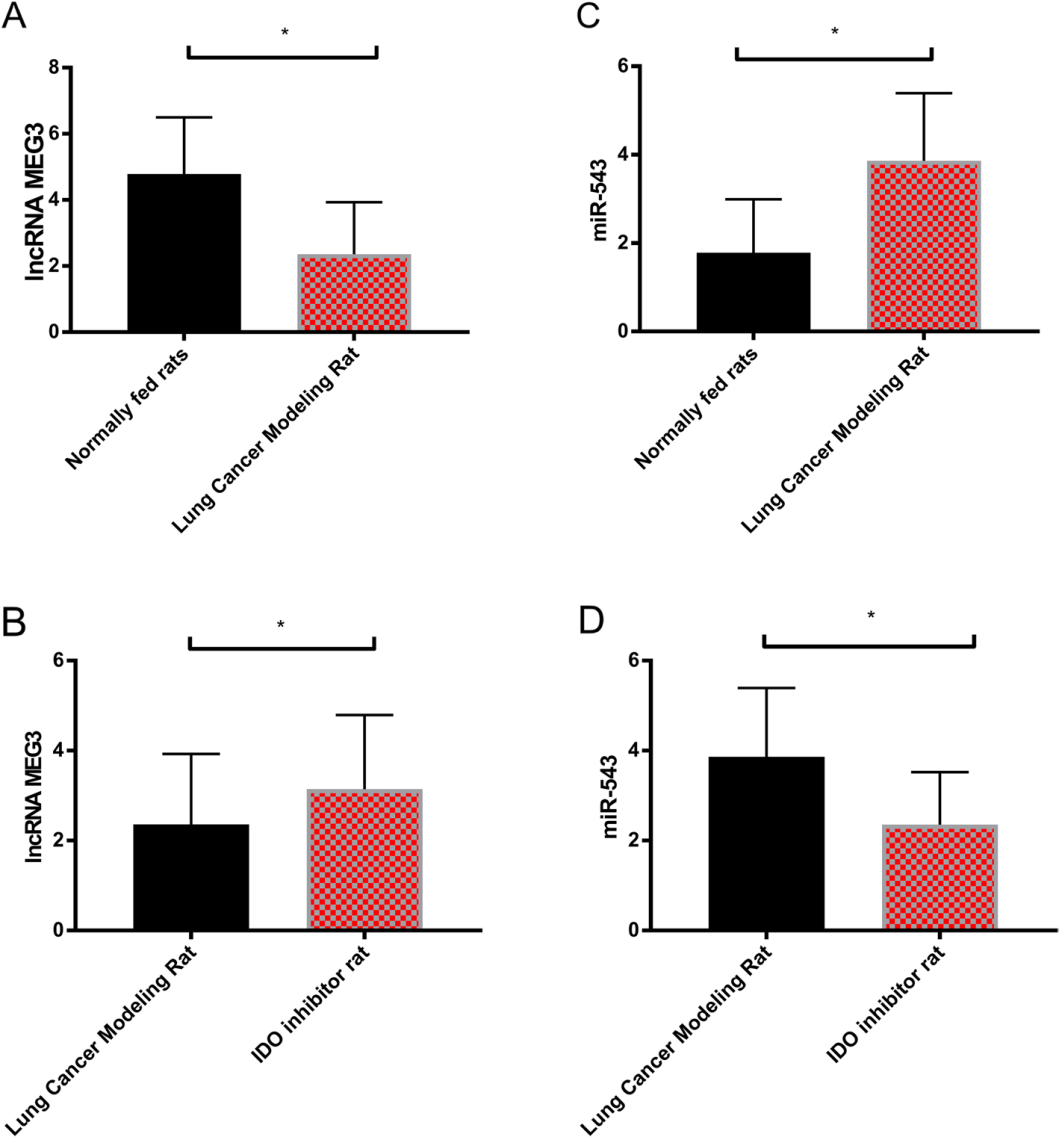
Expression of LncRNA MEG3 and miR-543 in lung tissue of three groups of rats. **A** LncRNA MEG3 expression in normally fed rats and lung carcinoma model rats. **B** LncRNA MEG3 expression in lung carcinoma model rats and IDO inhibitor rats. **C** miR-543 expression in normally fed rats and lung carcinoma model rats. **D** miR-543 expression in lung carcinoma model rats and IDO inhibitor rats

**Fig. 6:**
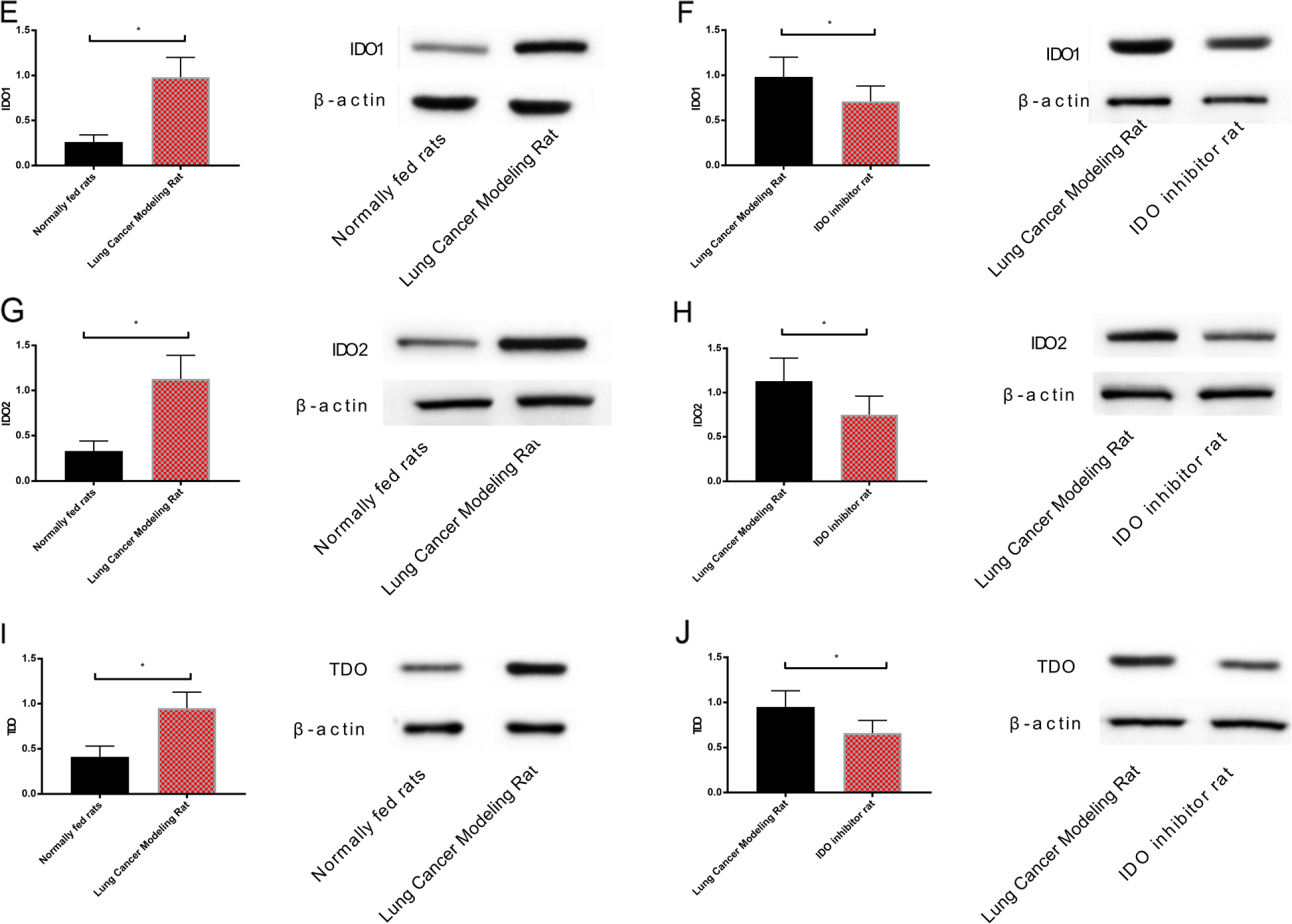
Expression of IDO (IDO1, IDO2, and TDO proteins) in lung tissue of three groups of rats. **E** Expression of IDO1 in normally fed rats and lung carcinoma model rats. **F** Expression of IDO1 in lung carcinoma model rats and IDO inhibitor rats. **G** Expression of IDO2 in normally fed rats and lung carcinoma model rats. **H** Expression of IDO2 in lung carcinoma model rats and IDO inhibitor rats. **I** TDO expression in normally fed rats and lung carcinoma model rats. **J** TDO expression in lung carcinoma model rats and IDO inhibitor rats

The level of LncRNA MEG3 in the lung tissue of normally fed rats was elevated compared with that of the lung carcinoma model (*P* < 0.05), and that of rats using IDO inhibitor was also evidently elevated compared with that of the lung carcinoma model (*P* < 0.05). Compared with lung carcinoma model rats, miR-543 in the lung tissue of normally fed rats and IDO inhibitor–intervened rats evidently declined (*P* < 0.05). Compared with lung carcinoma model rats, IDO1, IDO2, and TDO protein levels in the lung tissue of normally fed rats and IDO inhibitor–intervened rats reduced evidently (*P* < 0.05) (Figs. 5 and 6).

### Detection of immune function in lung tissue of three groups of rats

**Fig. 7:**
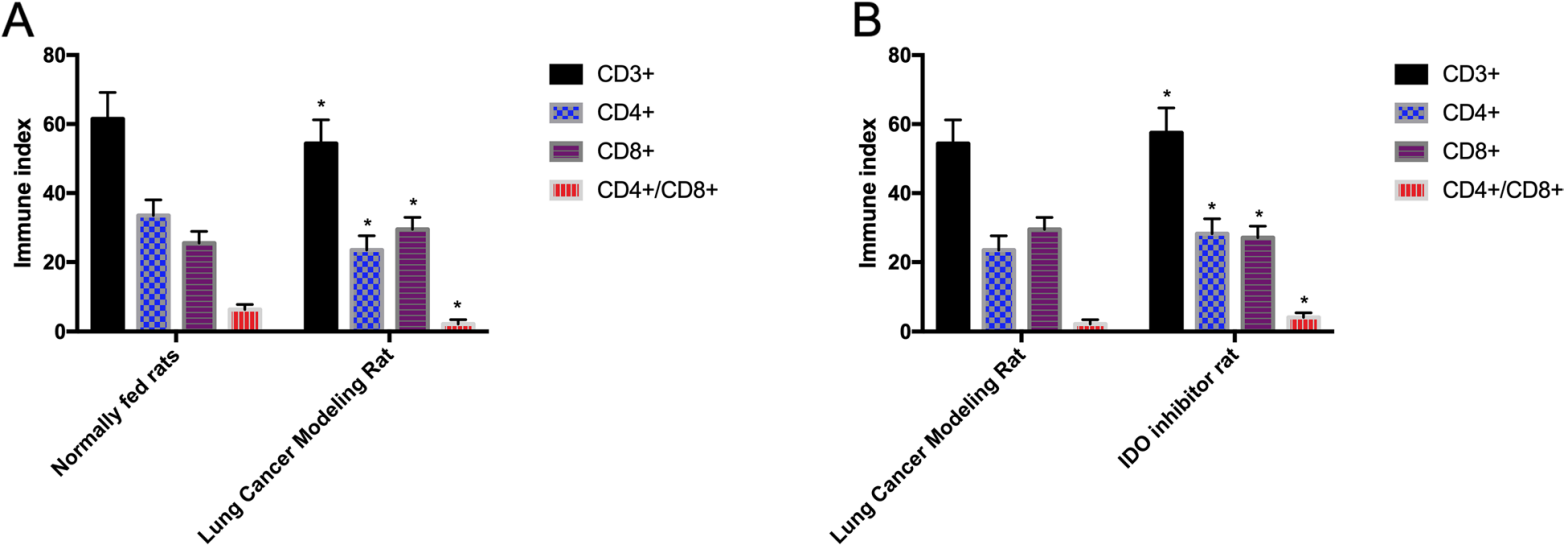
Immune function in lung tissue of three groups of rats. **A** Comparison of immune indexes in lung tissues of normally fed rats and lung carcinoma model rats. **B** Comparison of immune indexes between lung carcinoma model rats and IDO inhibitor rats

Immune indexes CD3^+^, CD4^+^, CD8^+^, and CD4^+^/CD8^+^ in the lung tissue of normally fed rats and lung carcinoma model rats were detected. The results showed that CD3^+^, CD4^+^, CD4^+^/CD8^+^ in normally fed rats were elevated compared with those in lung carcinoma model rats, while CD8^+^ was declined (*P* < 0.05) (Fig. 7).

### Role of LncRNA MEG3 on the biological behavior of NSCLC cells

**Fig. 8:**
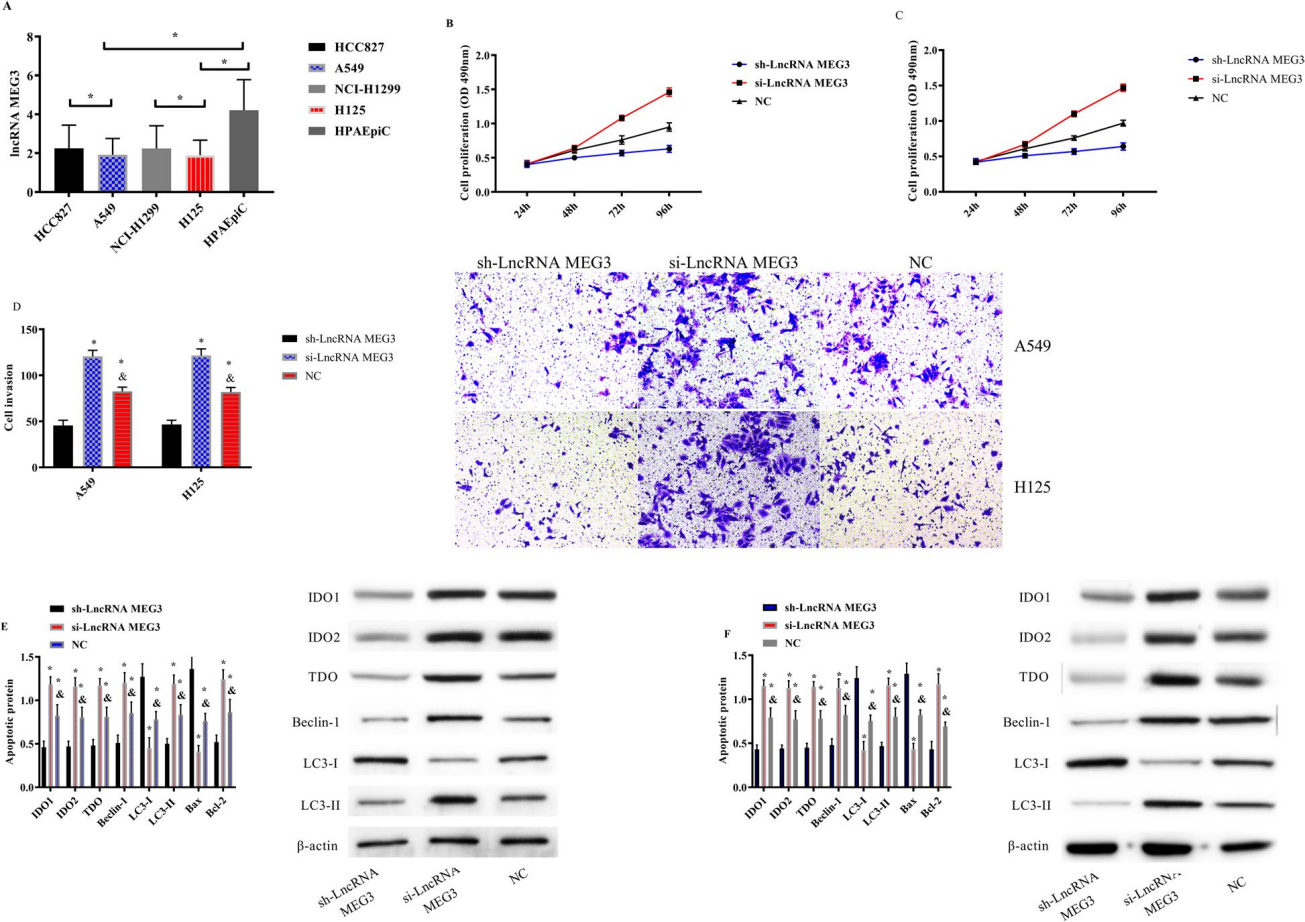
Effect of LncRNA MEG3 on biological behavior of NSCLC cells. **A** LncRNA MEG3 expression in different NSCLC cells. **B** Proliferation of A549 cells after transfection of LncRNA MEG3. **C** Proliferation of H125 cells after transfection of LncRNA MEG3. **D** Invasion of A549 and H125 cells after transfection of LncRNA MEG3. **E** Protein expression and Western Blot diagram of A549 cells after transfection of LncRNA MEG3. **F** Protein expression and western blot diagram of H125 cells after transfection of LncRNA MEG3. Note: Asterisk (*) indicates comparsion with the sh-LncRNA MEG3 group, and ampersand (&) indicates comparison with the si-LncRNA MEG3 group, *P* < 0.05)

LncRNA MEG3 in HCC827, A549, NCI-H1299, H125, and human alveolar epithelial cells HPAEpiC was tested. LncRNA MEG3 in NSCLC cells was lower (*P* < 0.05), among which the expression levels in A549 and H125 were the lowest (*P* < 0.05); so, A549 and H125 were selected for subsequent experiments. Transfection of LncRNA MEG3 into A549 and H125 showed that cell proliferation and invasion ability and IDO1, IDO2, TDO, Beclin-1, and LC3-II proteins in the sh-LncRNA MEG3 group were evidently declined compared with those in the NC group and the si-LncRNA MEG3 group, while the LC3-I protein was evidently elevated (*P* < 0.05). The proliferation; invasion; and DO1, IDO2, TDO, Beclin-1, and LC3-II proteins were the lowest in the sh-LncRNA MEG3 group, while the LC3-I protein was the highest (*P* < 0.05) (Fig. 8).

### Effect of miR-543 on the biological behavior of NSCLC cells

**Fig. 9:**
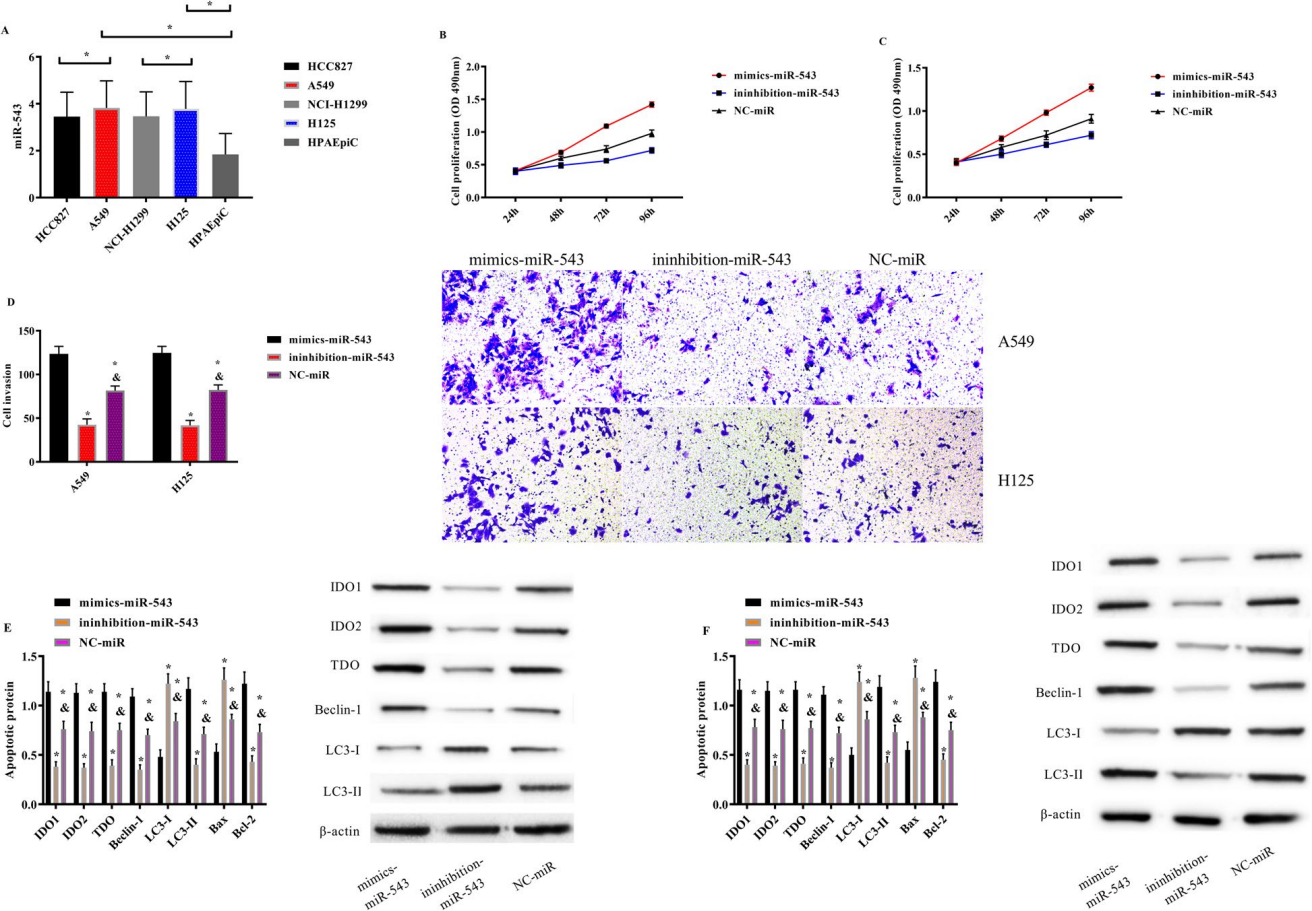
Effects of miR-543 on biological behavior of NSCLC cells. **A** miR-543 expression in different NSCLC cells. **B** Proliferation of A549 cells after transfection of miR-543. **C** Proliferation of H125 cells after transfection of miR-543. **D** Invasion of A549 and H125 cells after transfection of miR-543. **E** Protein expression and western blot diagram of A549 cells after transfection of miR-543. **F** Protein expression and western blot diagram of H125 cells after transfection of miR-543. Note: Asterisk (*) indicates comparison with the mimics-miR-543 group, and ampersand (&) indicates comparison with the inhibition-miR-543 group, *P* < 0.05

miR-543 in HCC827, A549, NCI-H1299, H125, and human alveolar epithelial cells HPAEpiC was detected, and miR-543 was high in NSCLC cells (*P* < 0.05). After transfection of miR-543 into A549 and H125 cells, follow-up experiments were carried out to test the changes in cell biological behavior. It was revealed that the proliferation and invasion and IDO1, IDO2, TDO, Beclin-1, and LC3-II proteins in mimics-miR-543 group were evidently elevated compared with those in inhibition-miR-543 group and NC-miR group, while the LC3-I protein was evidently declined (*P* < 0.05) (Fig. 9).

### Role of LncRNA MEG3, miR-543, and IDO in NSCLC

#### Correlation of LncRNA MEG3 with miR-543

**Fig. 10:**
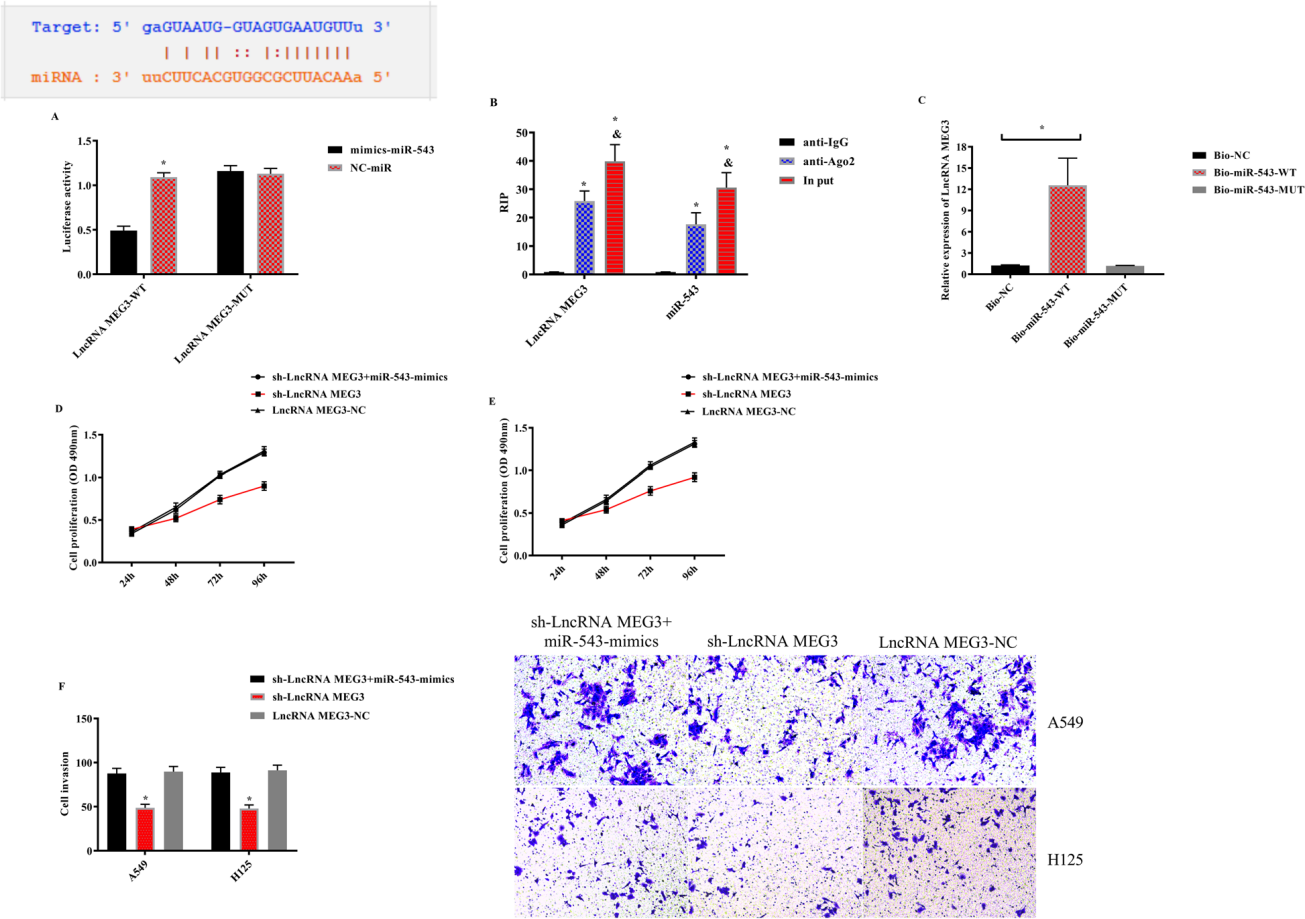
Relationship between LncRNA MEG3 and miR-543. **A** Double-fluorescent reporter assay, **B** RIP experiment, **C** RNA pull-down experiment, **D** proliferation of A549 cells, **E** proliferation of H125 cells, and **F** cell invasion

There was a latent binding target of LncRNA MEG3 with miR-543. The correlation of LncRNA MEG3 with miR-543 was further verified using double-fluorescence reporter assay, RIP, and RNA pull-down experiments. The results revealed that the fluorescence activity of LncRNA MEG3-WT was obviously hindered by mimics-miR-543, while LncRNA MEG3 and miR-543 precipitated by Ago2 antibody were evidently elevated compared with those of IgG; LncRNA MEG3 was pulled down by biotin-labeled miR-543-WT, but not by miR-543-MUT. Furthermore, LncRNA MEG3 was co-transfected with miR-543 to detect its biological function. The results revealed that there was no difference in cell proliferation and invasion between the sh-LncRNA MEG3 + miR-543-mimics group and LncRNA MEG3-NC group (*P* > 0.05) (Fig. 10).

#### LncRNA MEG3 influenced autophagy of lung carcinoma cells by regulating the miR-543/IDO signaling pathway

**Fig. 11:**
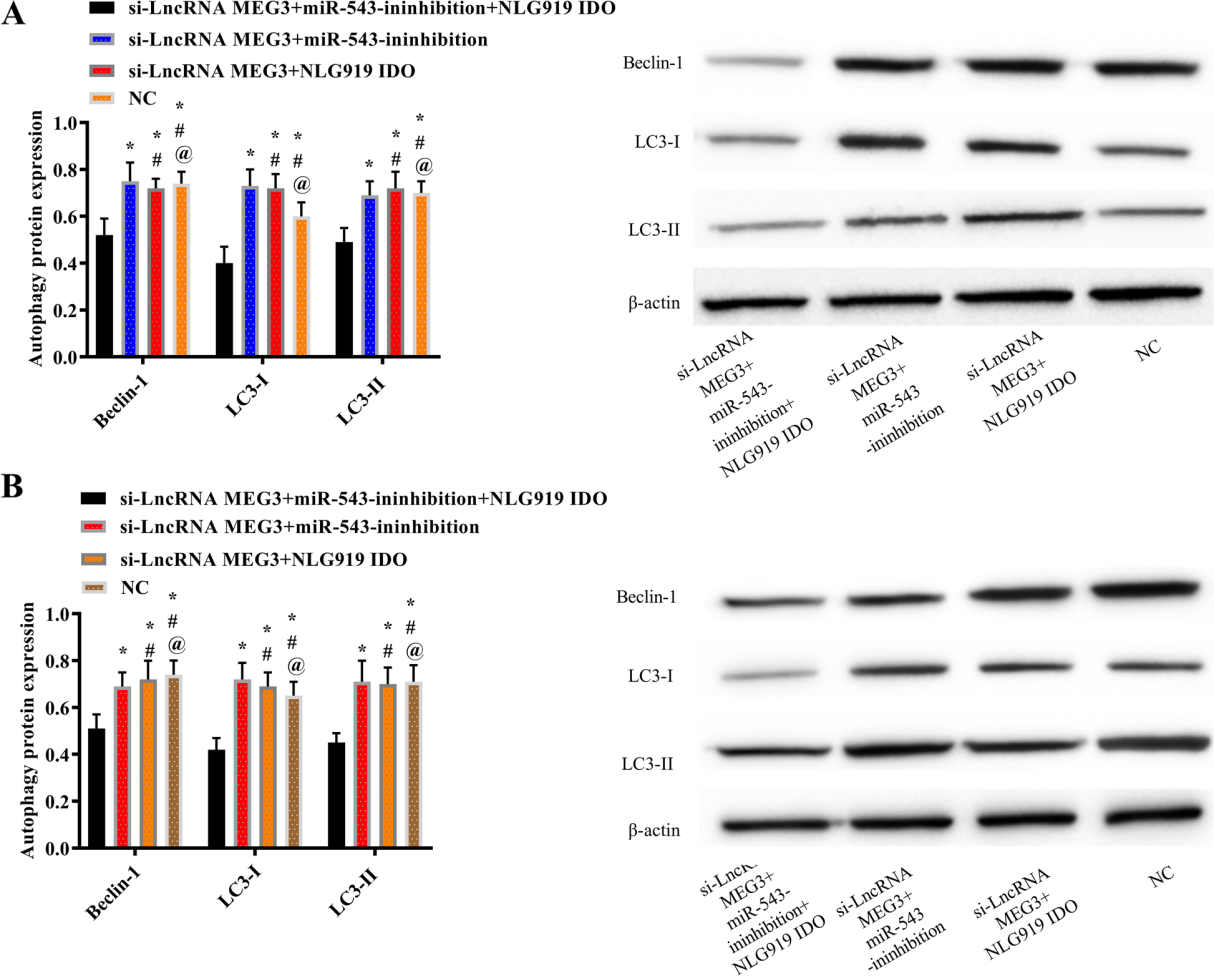
LncRNA MEG3 influenced autophagy of lung carcinoma cells by regulating miR-543/IDO signaling pathway. **A** Autophagy protein expression and western blot diagram of A549 cells. **B** Autophagy protein expression and western blot diagram of H125 cells. Note: Asterisk (*) indicates comparison with the si-LncRNA MEG3 + miR-543-ininhibition + NLG919 IDO group, number sign (#) indicates comparison with the si-LncRNA MEG3 + miR-543-ininhibition group, and @ indicates comparison with the si-LncRNA MEG3 + NLG919 IDO group, *P* < 0.05

Lung carcinoma cells were treated with si-LncRNA MEG3, miR-543-inhibition, and IDO signaling pathway inhibitor NLG919, and the cells were grouped into the si-LncRNA MEG3 + miR-543-inhibition + NLG919 IDO group, the si-LncRNA MEG3 + miR-543-inhibition group, the si-LncRNA MEG3 + NLG919 IDO group, and the NC group. Detection of autophagy protein expression in each group showed that there was no difference in the autophagy ability between the si-LncRNA MEG3 + miR-543-inhibition group, the si-LncRNA MEG3 + NLG919 IDO group, and the NC group (*P* > 0.05), which was elevated compared with the si-LncRNA MEG3 + miR-543-inhibition + NLG919 IDO group (Fig. 11).

## Discussion

Lung carcinoma is a common malignancy and the main cause of carcinoma-correlated mortality [16]. NSCLC is a kind of lung carcinoma, which causes almost 80% of lung carcinoma-related deaths [7]. NSCLC can be grouped into lung adenocarcinoma (LUAD), lung squamous cell carcinoma (LUSC), and large cell carcinoma (LC). Most cases are confirmed as incurable [8]. The treatment options include surgery, chemotherapy, radiotherapy, and targeted therapy [17]. In recent years, the biological behavior of NSCLC and molecular targeted therapy for the treatment have achieved encouraging results [18–20], but the prognosis of patients is still poor. The 5-year survival is only 10–15% [21]. The molecular mechanism is unclear. Hence, the molecular mechanism of NSCLC was investigated.

The experimental results revealed that LncRNA MEG3 was low in carcinoma tissues and peripheral blood, while miR-543 was high, suggesting that LncRNA MEG3 and miR-543 participate in the progression of NSCLC. Authors Wei et al. and Du et al. explored LncRNA MEG3 in gastric carcinoma and miR-543 in prostate carcinoma [22, 23]. The ROC curve revealed that the sensitivity and specificity of LncRNA MEG3 for predicting the occurrence of NSCLC were 60.87% and 82.05%, and those of miR-543 were 95.65% and 58.97%. Both have good diagnostic efficacy, suggesting that LncRNA MEG3 and miR-543 can be applied as tumor markers. Compared with traditional markers, LncRNA MEG3 and miR-543 can make up for their shortcomings, and assist in the early diagnosis and treatment of NSCLC, thus improving the prognosis of patients. In addition, by analyzing the correlation between LncRNA MEG3 and miR-543, we found that the two were negatively correlated. The results suggested that LncRNA MEG3 and miR-543 were closely related to NSCLC and that LncRNA MEG3 was related to miR-543. LncRNA MEG3 and miR-543 were detected before and after treatment in the RG. It was found that LncRNA MEG3 increased evidently, and miR-543 decreased evidently after treatment, which further supported our above experiments. In addition, we found that LncRNA MEG3 and miR-543 act on the prognosis of NSCLC patients, suggesting that LncRNA MEG3 and miR-543 can be applied as clinical screening indicators. Therefore, we purchased 40 rats for modeling, and succeeded in 38 rats, including 20 rats fed normally, 19 rats for lung carcinoma modeling, and 19 rats using IDO inhibitors. The expression of LncRNA MEG3, miR-543, and IDO (IDO1, IDO2, and TDO proteins) in the lung tissue of rats using IDO inhibitor was also evidently elevated, while miR-543 was declined compared with that of lung carcinoma model rats, suggesting that IDO inhibitor can obviously reduce the activity of carcinoma cells. Liu et al. proposed that IDO inhibitor can inhibit the proliferation of colon carcinoma cells [24], which is similar to our results. The immune function in lung tissues of the three groups of rats showed that CD3^+^, CD4^+^, CD4^+^/CD8^+^ of normally fed rats were higher, and CD8^+^ was declined compared with that of lung carcinoma model rats; and CD3^+^, CD4^+^, CD4^+^/CD8^+^ of IDO inhibitor rats were higher, and CD8^+^ was declined compared with that of lung carcinoma model rats. This suggested that IDO inhibitor can prevent this immunosuppression. We speculated that it will be a new research hotspot in NSCLC in the future as a new class of immunotherapy anti-carcinoma agents. Then, we transfected LncRNA MEG3 and miR-543 into NSCLC cells to detect their biological behavior and found that the proliferation and invasion ability, IDO1, IDO2, TDO, and autophagy-related Beclin-1 and LC3-II proteins of NSCLC cells overexpressing LncRNA MEG3 decreased evidently, while the autophagy-related LC3-I protein increased evidently, suggesting that LncRNA MEG3 acts as a tumor suppressor gene in NSCLC. Dong et al. [25] explored the mechanism of LncRNA MEG3 on esophageal carcinoma and obtained similar results. The influence of LncRNA MEG3 on NSCLC is still unclear. Previous explorations suggested that miR-543 was abnormally expressed in many tumor diseases [26–28], and the above experimental findings were closely related to NSCLC, so miR-543 in NSCLC cells was consistent with the above experimental results. In addition, the proliferation, invasion ability, IDO1, IDO2, TDO, autophagy-related Beclin-1 and LC3-II proteins of miR-543 group were evidently increased, while the autophagy-related LC3-I protein was evidently decreased. According to the above experiments, we speculated that there was a certain correlation of LncRNA MEG3 with miR-543, which was later confirmed by double fluorescent reporter assay. RIP and RNA pull-down tests revealed that LncRNA MEG3 and miR-543 precipitated by Ago2 antibody were evidently elevated compared with those of IgG. LncRNA MEG3 was pulled down by biotin-labeled miR-543-WT, but miR-543-MUT could not pull down LncRNA MEG3. The above experiments showed that LncRNA MEG3 can be applied as ceRNA to regulate miR-543. Furthermore, LncRNA MEG3 was co-transfected with miR-543 for biological function detection. There was no difference in cell proliferation, invasion between the sh-LncRNA MEG3 + miR-543-mimics group and the LncRNA MEG3-NC group, indicating that the changes of LncRNA MEG3 and miR-543 could affect the biological behavior of NSCLC cells. At last, we treated lung carcinoma cells with inhibition of LncRNA MEG3, inhibition of miR-543, and inhibitor of the IDO signaling pathway NLG919, respectively, and detected the autophagy protein expression of each group. It was found that there was no difference in autophagy ability among the inhibition of LncRNA MEG3 + inhibition of miR-543, the inhibition of LncRNA MEG3 + NLG919 IDO, and the NC groups. They were elevated compared with those in the LncRNA MEG3 + miR-543 + NLG919 IDO group. It was suggested that LncRNA MEG3 can affect lung carcinoma cells and autophagy by regulating the miR-543/IDO signaling pathway. Wang et al. [29] proposed that LncRNA MEG3 can promote the proliferation of ovarian carcinoma through PTEN, while Dan et al. [30] suggested that LncRNA MEG3 can hinder the proliferation and metastasis of gastric carcinoma by regulating miR-21, which also confirmed the consistent mechanism of action of LncRNA MEG3 in several tumor diseases. Further study on the influence of LncRNA MEG3 may be remarkable for the treatment of NSCLC and different malignancies.

This research intended to investigate the role of LncRNA MEG3 on NSCLC. There are still some limitations. For instance, the data of other types of lung carcinoma were not obtained, so we were unable to find the specific role of LncRNA MEG3 in other types of lung carcinoma. In addition, we did not know the effect of LncRNA MEG3 on the long-term prognosis. Moreover, we failed to conduct drug resistance and tumor formation tests in nude mice. We will conduct a more in-depth and detailed exploration to improve this.

## Conclusions

MEG3 is low expressed in NSCLC, and has an impact on the immunity and autophagy of NSCLC cells via regulating the miR-543/IDO signaling pathway, which is effective for the treatment of NSCLC.

## Data Availability

The datasets used and/or analyzed during the current study are available from the corresponding author on reasonable request.
